# External validation of the American prediction model for incident type 2 diabetes in the Iranian population

**DOI:** 10.1186/s12874-023-01891-y

**Published:** 2023-03-29

**Authors:** Samaneh Asgari, Davood Khalili, Fereidoun Azizi, Farzad Hadaegh

**Affiliations:** 1 Prevention of Metabolic Disorders Research Center, Research Institute for Endocrine Sciences, Shahid Beheshti University of Medical Sciences, Tehran, Iran; 2 Department of Biostatistics and Epidemiology, Research Institute for Endocrine Sciences, Shahid Beheshti University of Medical Sciences, Tehran, Iran; 3 Endocrine Research Center, Research Institute for Endocrine Sciences, Shahid Beheshti University of Medical Sciences, Tehran, Iran

**Keywords:** Type 2 diabetes, Prediction model, External Validation

## Abstract

**Background:**

The primary aim of the present study was to validate the REasons for Geographic and Racial Differences in Stroke (REGARDS) model for incident Type 2 diabetes (T2DM) in Iran.

**Methods:**

Present study was a prospective cohort study on 1835 population aged ≥ 45 years from Tehran lipids and glucose study (TLGS).The predictors of REGARDS model based on Bayesian hierarchical techniques included age, sex, race, body mass index, systolic and diastolic blood pressures, triglycerides, high-density lipoprotein cholesterol, and fasting plasma glucose. For external validation, the area under the curve (AUC), sensitivity, specificity, Youden’s index, and positive and negative predictive values (PPV and NPV) were assessed.

**Results:**

During the 10-year follow-up 15.3% experienced T2DM. The model showed acceptable discrimination (AUC (95%CI): 0.79 (0.76–0.82)), and good calibration. Based on the highest Youden’s index the suggested cut-point for the REGARDS probability would be ≥ 13% which yielded a sensitivity of 77.2%, specificity 66.8%, NPV 94.2%, and PPV 29.6%.

**Conclusions:**

Our findings do support that the REGARDS model is a valid tool for incident T2DM in the Iranian population. Moreover, the probability value higher than the 13% cut-off point is stated to be significant for identifying those with incident T2DM.

## Background

The prevalence and incidence of type 2 diabetes (T2DM) are increasing in developed and developing countries as well. According to the International diabetes federation (IDF) report, in 2019 more than 460 million adults (20–79 years) were living with diabetes and it is expected to increase to 700 million by 2045 [1]. According to these reports, about 80% of adults were living in low-and middle-income countries, but only 35% of health expenditure on diabetes was spent there. Therefore, early identification of those at high risk of T2DM, specifically in low and middle-income countries is an important health concern.

During the last 20 years, different risk prediction models were developed for detecting incident T2DM [2, 3] and the majority of them use logistic or survival regression. Lotfaliani.M et al. [4, 5] validated several clinical and lab-based prediction models such as FINDRISC (Finnish Diabetes Risk Score) [6], AUSDRISK (Australian Type 2 Diabetes Risk Assessment Tool) [7], Framingham Offspring Study (FOS) [8] and ADA (American Diabetes Association Risk Score) [9] for identifying T2DM among Iranian population; the minimum value of the area under the curve (AUC) of these models was 0.7 and family history of diabetes (FH-DM) was the main risk factor of all these risk prediction models [4, 5].

In 2020 a new model was suggested by Wilkinson et al. [10] for the 10-year prediction of incident T2DM in the American population using REasons for Geographic And Racial Differences in Stroke (REGARDS) data. The main difference between the new models from the previous ones lies in its methodology which aimed to consider sex and race differences using Bayesian logistic regression. Despite the good discrimination of the above model, since study participants were only non-Hispanic white or black and family history of diabetes (FH-DM) was not included in the model calculation, the generalizability of the results has been questioned [11, 12].

Although in the same paper the author externally validated the introduced model in the American population using the ARIC data (Atherosclerosis Risk in Communities study), it was not validated in other populations. Because of different race/ethnicity, behavioral, and biological factors, the performance of screening tools for incident T2DM could be different among populations [13] and the generalizability of the introduced model must be validated in local populations [14]. Therefore, in the present study, considering the above concerns, we first validated the REGARDS model in a large external cohort of Iranian. We, further, aimed to assess different cut-offs for REGARDS probability, as it was not considered by Wilkinson et al. [10].

## Materials and methods

### Study population

Tehran Lipid and Glucose Study (TLGS) is a community-based prospective cohort study conducted on an Iranian urban population in Tehran. The study aims to determine the prevalence and incidence of non-communicable diseases and related risk factors among individuals aged ≥ 3 years and promote a healthy lifestyle and programs for the prevention of non-communicable diseases (NCDs). The study has been established in the first phase (1999–2001: n = 15,005) and is planned to keep on for at least 20 years on a triennial basis (i.e., second phase: 2001–2005, third phase: 2005–2008, fourth phase: 2009–2011, fifth phase: 2012–2015, and sixth phase: 2015–2018). The design and methodology of the TLGS study have been reported elsewhere [15].

**Fig. 1:**
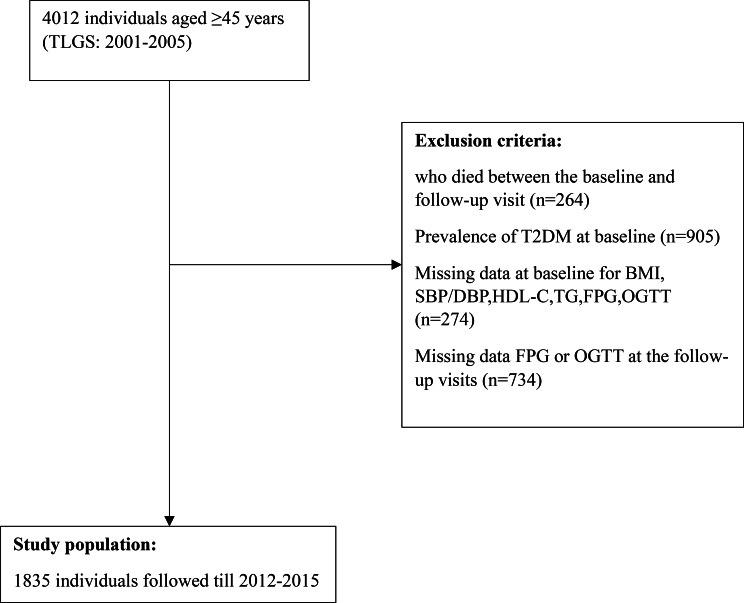
Flow diagram of the study participants Body mass index (BMI), systolic/diastolic blood pressure (SBP/DBP), high-density lipoprotein cholesterol (HDL-C), triglycerides (TG), fasting plasma glucose (FPG), and oral glucose tolerance test (OGTT); TLGS: Tehran lipids and glucose study

For the current study, phase 2 (2001–2005) of the TLGS was considered as the baseline. By following the same procedure as that reported by Wilkinson et al. [10], from 4012 individuals aged ≥ 45 years, we excluded those who died between the baseline and follow-up visit (n = 264) and those with the prevalence of T2DM at baseline (n = 905). We further excluded those with missing data (complete case analysis) at baseline for body mass index (BMI), systolic/diastolic blood pressure (SBP/DBP), high-density lipoprotein cholesterol (HDL-C), triglycerides (TG), fasting plasma glucose (FPG), and oral glucose tolerance test (OGTT) (n = 274) as well as FPG or OGTT at the follow-up visits (n = 734). Finally, 1835 (response rate: 56.5%) individuals who had information on the fifth phase (2012–2015) were eligible for the current study (Fig. 1).

This study was performed by the Declaration of Helsinki and was approved by the Institutional Review Board (IRB) of the Research Institute for Endocrine Sciences (RIES), Shahid Beheshti University of Medical Sciences, Tehran, Iran, and all participants provided written informed consent. We also confirmed that all methods were performed by the relevant guidelines and regulations.

### Clinical and laboratory measurements

Information on demographic data and medication history was obtained by a trained interviewer using a standard questionnaire. Details for anthropometric measurements including height and weight were reported elsewhere [15]. Measurements of SBP and DBP were taken on the right arm after a 15-min rest in a sitting position. A blood sample was taken from all study participants between 7:00 and 9:00 AM after 12 to 14 h of overnight fasting. More detail for laboratory measurements including FPG, OGTT, HDL-C, and TG was addressed previously [15].

### Definition of T2DM

Diabetes was defined as having FPG ≥ 7 mmol/L and/or OGTT ≥ 11.1 mmol/L, using anti-diabetic medications, or self-reported T2DM [16].

### REGARDS model

Wilkinson et al. [10] suggested the 10-year prediction risk score for incident T2DM in the American population using REGARDS data. Age, sex, BMI, SBP, DBP, HDL-C, TG, FPG, and race were risk factors for incident T2DM which were included in the risk calculation using Bayesian hierarchical techniques.

$$\begin{array}{c}score = - 8.464 - 0.014 \times age + 0.053 \times BMI + \\0.006 \times SBP + 0.003 \times DBP + 0.062 \times \\FPG - 0.018 \times HDL - C + 0.001 \times \\TG - 0.084 \times Sex\left( {women{\mkern 1mu} as{\mkern 1mu} reference} \right)\\- 0.466 \times Race\left( {black{\mkern 1mu} as{\mkern 1mu} reference} \right)\end{array}$$

Moreover, the predictive risk probabilities for any participants can be calculated using the following function:

$$Probability={logit }^{-1}\left(score\right)$$

$$=\frac{\text{exp}\left(score\right)}{[1+\text{exp}\left(score\right)]}$$

### Statistical analysis

Baseline characteristics of the study population were expressed as mean (standard deviation:SD) and number (%) for categorical variables. For covariates with a skewed distribution (e.g. TG and follow-up duration), the median (interquartile range: IQR) was reported. A comparison of baseline characteristics between those with and without T2DM as well as responders (study population) and non-responders (those with missing values or without any follow-up data) was done by the Student’s t-test for normally distributed continuous variables, Mann-Whitney u test for skewed variables, and the chi-squared test for categorical variables.

To evaluate the external validity of the risk equation, the area under the receiver operating characteristic curve (AUC) was applied to determine the discrimination ability. According to the Hosmer et al. [17] criteria, the AUCs 0.5–0.7, 0.70–80, 0.80–0.90, and ≥ 0.90 indicated poor, acceptable, excellent, and outstanding discrimination, respectively. To show the calibration in detail, the observed risk was plotted versus the mean of predicted probabilities over deciles. Validation of the REGARDS model was done using sensitivity, specificity, positive and negative predictive value (PPV and NPV), Youden’s index (sensitivity + specificity-1), positive likelihood ratio (LR+; sensitivity/(1 − specificity)), and negative likelihood ratio (LR−;((1 − sensitivity)/specificity)). We also estimated the cut-off for the REGARDS score that would result in the highest AUC when applied in the TLGS cohort and compared the performance with the recommended risk discussion cut-points for diabetes ≥ 10%, ≥ 20%, and ≥ 30% [18]. The suggested cut-off point was calculated based on the highest value of Youden’s index. Statistical analysis was performed using STATA version 16 (StataCorp LP, College Station, Texas), statistical software. P  ≤0.05 were considered statistically significant.

## Results

The study population consisted of 1,835 (men = 837) with a mean (SD) age of 56.02(7.89) years. The baseline characteristics of those with and without T2DM are shown in Table 1. There were significant differences between those with and without T2DM; they had higher levels of BMI, WC, SBP, DBP, TG, HDL-C, and FPG, and had a higher percentage of FH-DM than those without T2DM. Among responders and non-responders, a few differences were observed in which responders were younger and had lower levels of SBP (Table 2).

**Table 1:** Baseline characteristics of the study population: Tehran Lipid and glucose study

	Total (N = 1835)	With T2DM (N = 281)	Without T2DM (N = 1554)	P-value
Sex, men	837(45.6)	123(43.8)	714(45.9)	0.5
Age, (years)	56.02(7.89)	56.5(7.6)	55.9(7.9)	0.22
Body mass index, (kgm2)	28.27(4.38)	29.9(4.6)	28.0(4.3)	< 0.001
Waist circumference, (cm)	95.21(10.33)	99.6(10.2)	94.4(10.2)	< 0.001
Systolic blood pressure, (mmHg)	122.76(18.25)	127.9(17.4)	121.8(18.3)	< 0.001
Diastolic blood pressure, (mmHg)	77.34(10.47)	79.7(10.6)	76.9(10.4)	< 0.001
Fasting plasma glucose, (mmol/L)	5.15(0.54)	5.62(0.59)	5.06(0.48)	< 0.001
High density lipoprotein cholesterol, (mmol/L)	1.02(0.27)	0.98(0.26)	1.03(0.27)	0.006
Triglycerides, (mmol/L)	1.72(1.12)	2.02(1.37)	1.64(1.05)	< 0.001
Family history diabetes, (yes)	249(13.6)	56(19.9)	193(12.4)	0.001
Follow-up duration, (years)	9.31(1.65)	9.4(1.71)	9.3(1.62)	0.17

Data are shown as mean (SD) for continues and number (%) for categorical covariates; IQR: Interquartile range.SD: standard deviation; IQR: interquartile range

**Table 2:** Baseline characteristics of the responders (study population) and non-responders: Tehran Lipid and glucose study

	Responders (N = 1835)	Non-responders (N = 1008)	P-value
Sex, men	837(45.6)	466(46.2)	0.75
Age, (years)	56.02(7.89)	60.17(10.10)	< 0.0001
Body mass index, (kgm2)	28.27(4.38)	27.93(5.04)	0.07
Waist circumference, (cm)	95.21(10.33)	95.38(11.12)	0.69
Systolic blood pressure, (mmHg)	122.76(18.25)	127.4(21.55)	< 0.0001
Diastolic blood pressure, (mmHg)	77.34(10.47)	77.55(11.54)	0.62
Fasting plasma glucose, (mmol/L)	5.15(0.54)	92.96(10.07)	0.71
High density lipoprotein cholesterol, (mmol/L)	1.02(0.27)	1.03(0.27)	0.37
Triglycerides, (mmol/L)	1.72(1.12)	1.61(1.11)	0.12
Family history diabetes, (yes)	249(13.6)	125(12.4)	0.38
Follow-up duration, (years)	9.31(1.65)	9.32(1.76)	0.81

Data are shown as mean (SD) for continues and number (%) for categorical covariates; IQR: Interquartile range.SD: standard deviation; IQR: interquartile range

**Fig. 2:**
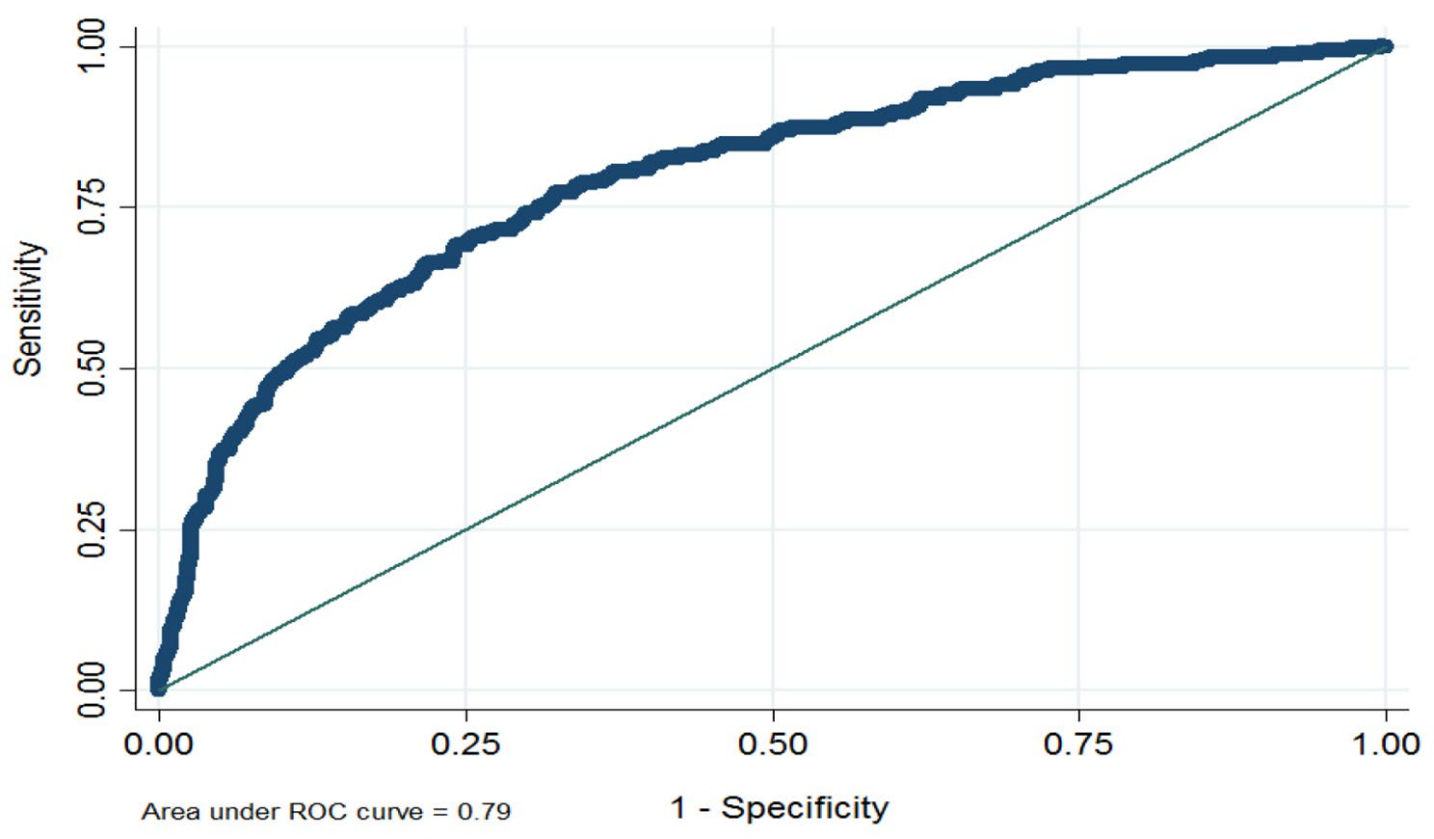
The area under the curve of REGARDS score for incident T2DM REGARDS: REasons for Geographic and Racial Differences in Stroke; T2DM: Type 2 diabetes

**Fig. 3:**
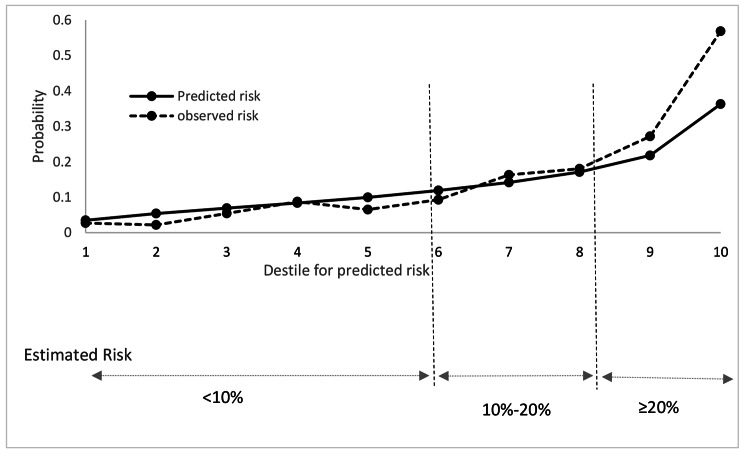
Predicted vs. observed risk of type 2 diabetes

During the median (IQR) follow-up of 9.3 (8.4–10.1) years, the cumulative incidence of T2DM among the whole population was 281(15.3%). As shown in Fig. 2, the discrimination power of the model calculated by AUC (95% CI) was 0.79 (0.76–0.82). The predicted vs. observed risk of T2DM was shown in Fig. 3. The REGARDS model shows good calibration, especially for those with a risk probability ≤ 20%.

The diagnostic characteristics of the REGARDS model were shown in Table 3. The probability threshold of ≥ 10% defined about 55% of the population as high-risk and yielded a sensitivity of 84.7%, specificity 50.9%, NPV 94.8%, and PPV 23.8%. Maximum Youden’s index indicated a threshold of ≥ 13%. Considering this cut point, about 40%of the total population was classified as high-risk individuals and resulting in a sensitivity of 77.2%, a specificity of 66.8%, PPV of 29.6%, and NPV of 94.2% with a positive LR of 2.33 and a negative LR of 0.34.

**Table 3:** The clinical performance of the REGARDS model for incident type 2 diabetes: Tehran Lipid and glucose study

Probability threshold (%)	≥ 10	≥ 13	≥ 20	≥ 30
Population aged ≥ 45 years (N = 1835, DM = 281)				
High-risk population, %	54.55	39.95	18.00	6.76
Incident diabetes*,%	23.8	29.6	44.24	62.10
Sensitivity, % (95% CI)	84.7 (79.9–88.7)	77.2 (71.9–82.0)	52.0 (45.9–57.9)	27.4 (22.3–33.0)
Specificity, % (95% CI)	50.9 (48.4–53.4)	66.8 (64.4–69.1)	88.2 (86.4–89.7)	97.0 (96.0-97.8)
PPV, % (95% CI)	23.8 (21.2–26.5)	29.6 (26.3–33.1)	44.2 (38.8–49.8)	62.1 (52.9–70.7)
NPV, % (95% CI)	94.8 (93.1–96.2)	94.2 (92.6–95.5)	91.0 (89.5–92.4)	88.1 (86.4–89.6)
LR+ (95% CI)	1.73 (1.61–1.85)	2.33 (2.12–2.56)	4.39 (3.68–5.23)	9.1 (6.5–12.7)
LR- (95% CI)	0.30 (0.23–0.40)	0.34 (0.27–0.42)	0.54 (0.48–0.62)	0.75 (0.70–0.80)
Youden Index, % (95% CI)	35.6 (28.3–42.1)	**44.0 (36.3–51.1)**	40.2 (32.3–47.6)	24.4 (18.3–30.8)
AUC (95% CI)	0.68 (0.65–0.70)	0.72 (0.69–0.75)	0.70 (0.67–0.73)	0.62 (0.60–0.65)

All analysis was done based on survey data analysis (weighted statistics); PPV: positive predictive value; NPV: negative predictive value; LR: Likelihood ratio.

*Incident diabetes was reported among the high-risk population.

## Discussion

The current study is the first independent external validation of a 10-year risk prediction model for incident T2DM using Bayesian logistic regression. The model showed acceptable discrimination and good calibration. We also showed that the probability threshold ≥ 13% has good power to classify the low and high-risk adults for incident T2DM.

Generally compared with the development data [10], our population is younger, with higher levels of SBP/DBP, and the same follow-up duration. The REGARDS model showed an acceptable discriminative performance with slightly higher AUC levels in the TLGS population (0.79) compared to the development data (0.789). As reported by Wilkinson et al. [10] the AUC was improved (0.85) when the model fitted using ARIC data. This difference might be explained by the difference in populations and study periods [19]. In the current study, we showed that the REGARDS prediction models provided valid calibration using the TLGS data, especially for those with a probability < 20%.

Several risk prediction models were validated and updated among the Iranian population [20, 21] (Table 4). Among lab-based prediction models which was validated by Lotfaliany et al. [5], ARIC (AUC (95% CI): 0.825(0.795–0.855)) risk prediction model had the highest discrimination compared with Saint Antonio (SA; 0.808(0.776–0.839)) and Framingham Offspring Study (FOS; 0.816(0.784–0.848)). They also showed that the original models of these three prediction models overestimated the risk; after re-calibration, ARIC, SA, and FOS show a good calibration. In another study, Lotfaliany et al. [4] validated and compared office-based risk prediction models including the Finnish Diabetes Risk Score (FINDRISC), the Australian Type 2 Diabetes Risk Assessment Tool (AUSDRISK), and the American Diabetes Association Risk Score (ADA) for undiagnosed and incident T2DM. During 5 years of follow-up AUSDRISK had the highest discrimination (0.767(0.747–0.787)) compared to FINDRISC (0.754(0.733–0.775)) and ADA (0.726(0.704-748)). Moreover the re-calibrated models for FINRISK and ADA and the original model of AUSDRISK showed good calibration. In addition to the external validation, Bozorgmanesh et al. [26] developed a simple risk score based on SBP, waist to height ratio, TG/HDL-C, FPG, and FH-DM to predict incident T2DM using TLGS study population. The AUC (95% CI) of the model was 0.83(0.80–0.86) with good calibration.

**Table 4:** Previous investigation on the external validation of prediction models for undiagnosed/ incident type 2 diabetes using Tehran Lipid and glucose study data

Prediction model	Population/year	Validation Data	Risk predictors in the final model	Statistical models	C-index/AUC	Calibration
San Antonio heart study diabetes prediction model [22]	Mexican Americans and Hispanic white/2010	TLGS cohort / the 6.3-year incidence of T2DM	age, sex, ethnicity, SBP, HDL-C, BMI, FH-DM, FPG	Logistic regression	0.83	Acceptable after recalibrated
The Saint Antonio Diabetes Prediction Model (SA) [23]	Mexican Americans and Hispanic white/2021	TLGS cohort / The 5-year incidence of T2DM	age, sex, ethnicity, SBP, HDL-C, BMI, FH-DM, FPG	Logistic regression	0.81	Acceptable after recalibrated
Atherosclerosis Risk in Communities Study (ARIC) [24]	American population/2013	TLGS cohort / The 6-year incidence of T2DM	Age, FH-DM, hypertension, WC, height TG, HDL-C, FPG, race	Cox regression	Men 0.790 Women 0.829	Acceptable after recalibrated
Atherosclerosis Risk in Communities Study (ARIC) [23]	American population/2021	TLGS cohort / The 5-year incidence of T2DM	Age, FH-DM, hypertension, WC, height TG, HDL-C, FPG, race	Logistic regression	0.83	Acceptable after recalibrated
Finnish Diabetes Risk Score (FINDRISC) (4)	Finish population/2019	TLGS cohort / For undiagnosed T2DM	age, BMI, WC, physical activity, daily consumption of fruits, berries, or vegetables, and the history of antihypertensive drug treatment and history of high blood glucose to predict drug-treated diabetes	Logistic regression	0.75	Acceptable after recalibrated
Australian Type 2 Diabetes Risk Assessment Tool (AUSDRISK) (4)	Australian population/2019	TLGS cohort / For undiagnosed T2DM	Non-invasive model: age, sex, ethnicity, FH-DM, history of high blood glucose level, use of antihypertensive medications, smoking, physical inactivity, and WC	Logistic regression	0.77	Acceptable calibration
Australian Type 2 Diabetes Risk Assessment Tool (AUSDRISK) for undiagnosed diabetes [23]	Australian population/2021	TLGS cohort / The 5-year incidence of T2DM	Invasive model: age, race, FH-DM, FPG, SBP, WC, height, HDL-C, and TG	Logistic regression	0.77	Acceptable after recalibrated
American Diabetes Association Risk Score (ADA) [4]	American population/2019	TLGS cohort/ For undiagnosed T2DM	Age, sex, FH-DM, history of hypertension, obesity, and physical activity	Logistic regression	0.73	Acceptable after recalibrated
risk assessment tool for cardiovascular disease, type 2 diabetes, and chronic kidney disease [25]	Dutch population/2020	TLGS cohort / For undiagnosed T2DM	Sex stratified analysis: age, BMI, WC, use of antihypertensive medications, current smoking, parent and/or sibling with MI or stroke (age < 65 years), FH-DM	Logistic regression	Men 0.65 Women 0.69	Not acceptable
American Diabetes Association screening tool [21]	American population/2020	national survey of risk factors for non-communicable diseases / For undiagnosed T2DM	Age, sex, FH-DM, history of hypertension, obesity, and physical activity	Logistic regression	0.737	Not reported
The Framingham Offspring Study (FOS) risk score [23]	American population/2021	TLGS cohort / The 5-year incidence of T2DM	age, gender, FPG, BMI, WC, HDL-C, SBP, FH-DM	Logistic regression	0.82	Acceptable after recalibrated
REasons for Geographic And Racial Differences in Stroke (REGARDS) / Current study	American population	TLGS cohort / The 10-year incidence of T2DM	Age, sex, BMI, SBP, DBP, HDL-C, TG, FPG, and race	Baysian logistic regression	0.79	Acceptable calibration

T2DM: type 2 diabetes; SBP: systolic blood pressure; HDL-C: high density lipoprotein cholesterol; TG: triglycerides; BMI: body mass index; WC: waist circumference; FH-DM: family history diabetes; FPG: fasting plasma glucose; MI: myocardial infarction.

Wilkinson et al. [10] did not suggest any cut-off point for estimated probability. However, using TLGS data, our recommended probability cut-point for the detection of incident T2DM is ≥ 13%. Considering a higher threshold of ≥ 15%, the sensitivity decreased to 69%, and the specificity increased to 75%. With lower sensitivity, we miss several adults with incident T2DM, while higher specificity increases the number of individuals for further identification tests. Therefore, from the public health point of view, the selection of the clinical cut-off point needs more caution.

The evidence shows that the well-known T2DM risk factors (e.g. age, sex, BMI, FPG, lipids, hypertension, and FH-DM) are commonly used in developing regression-based prediction models [27]. Most of these variables (except for FH-DM) were included in the REGARDS prediction model. According to the recently published systematic review on the prediction models for undiagnosed and incident T2DM, FH-DM was the main predictor in 47% and 31% of the models, respectively. However, FH-DM is subjective and non-quantitative but it was reported that the prevalence of diabetes among those who reported positive FH-DM was 14.94% (6.48% diabetic fathers and 10% diabetic mothers) [28]. Hariri et al. [29] reported that individuals with positive FH-DM compared with those without, have a higher perceived risk of diabetes. Additionally, they showed that a positive FH-DM was identified in 73% of individuals with T2DM and correctly predicted prevalent T2DM in 21.5%. According to the InterAct Consortium report [30], positive FH-DM increased the risk of incident T2DM more than 2.5 fold, whereas the range of missing information about this important risk factor was between 0.1% in the UK to 24% in Denmark (12% in France; 0.8% Netherlands; 13% Germany; 20% Sweden). The frequency of missing information on FH-DM in the national Iranian survey follows the WHO STEPwise approach to Surveillance in 2011 [21] for those aged ≥ 45 years (n = 4,325), was 2.6% (data not shown). Although due to the lack of information on the FH-DM for the REGARDS study it was not included in the prediction model, validation of the model in TLGS data was appropriate even without FH-DM.

This study had several strengths. Firstly, to the best of our knowledge, this is the first study in the Middle East and North Africa that validated this American model on the Iranian population. Secondly, unlike the original model, we suggested a threshold for a higher risk of incident T2DM. As a limitation, this study was done among the urban population of Tehran and the generalizability is not known for the rural population.

In conclusion, our findings do support that the REGARDS model is a valid tool for incident T2DM in the Iranian population. Moreover, the probability value higher than the 13% cut-off point is stated to be significant for identifying those with incident T2DM.

## Data Availability

The datasets generated and/or analyzed during the current study are not publicly available due to local data protection regulations but are available from the corresponding author at reasonable request.

## Abbreviations

REGARDS: REasons for Geographic and Racial Differences in Stroke
T2DM: Type 2 diabetes
FH-DM: Family history of diabetes
TLGS: Tehran lipids and glucose study
AUC: Area under the curve
IDF: International diabetes federation
ARIC: Atherosclerosis Risk in Communities study
NCDs: Non-communicable diseases
IQR: Interquartile range
BMI: Body mass index
SBP/DBP: Systolic/diastolic blood pressure
TG: Triglycerides
FPG: Fasting plasma glucose
OGTT: 2-hour post-challenge plasma glucose
O/E: Observed-expected ratio
DIC: Deviance information criterion
